# Handling COVID-19 in the capital city of Jakarta with innovation policy: the scale of social restrictions policy^☆^

**DOI:** 10.1016/j.heliyon.2022.e09467

**Published:** 2022-05-19

**Authors:** Retnowati WD. Tuti, Achmad Nurmandi, Abitassha Az Zahra

**Affiliations:** a Department of Public Administration, Universitas Muhammadiyah Jakarta, Indonesia; b Department of Government Affairs and Administration, Jusuf Kalla School of Government, Universitas Muhammadiyah Yogyakarta, Indonesia

**Keywords:** Innovation public policy, The scale of social restrictions, COVID-19

## Abstract

This study aimed to examine the dominant innovation policy elements of the social restrictions to fight the COVID-19 pandemic in Jakarta. The data was obtained using a Computer-Aided Qualitative Data Analysis Software named the NVivo 12 Plus application, accompanied by purposive sampling from four online media sources. These sites, namely Detik.com, Kompas.com, Liputan6.com, and Kumparan.com, were accessed for their provision of information about the COVID-19 social restriction policy in DKI Jakarta. Subsequently, the results showed that the Jakarta government's decision on the Large-Scale Social Restriction-PSBB Stage 1–5 and PPKM Stage consider relative advantage and trialability aspects. Following the proposed policy design, the Jakarta Government expects an easier adoption of the innovation in slowing the spread of the virus. Relative advantage and trialability were revealed to enable developing countries to manage or control the number of pandemic cases and the ensuing economic impact, as well as innovate their policies in practical cases based on the field situation. This study only focused on Jakarta as one of the South East capital cities that successfully dealt with COVID-19. Therefore, future studies should obtain policy designs from other continents that successfully tackled the pandemic in different situations.

## 1 Introduction

On January 30, 2020, the WHO declared COVID-19 a global health emergency and explained its rapid spread to other locations (Velavan and Meyer, 2020). Due to the major challenge caused by the health and social impact of the pandemic on communities and the government (Vecchio et al., 2020), the adoption of unprecedented policy designs has been promoted to slow the virus's growth rate (Hsiang et al., 2020). Countries have attempted innovation by formulating several strategies to slow the spread of COVID-19 (Liao et al., 2020) rather than developing easily-adopted policies that would be more important during this fast transmission pandemic.

**Figure 1:**
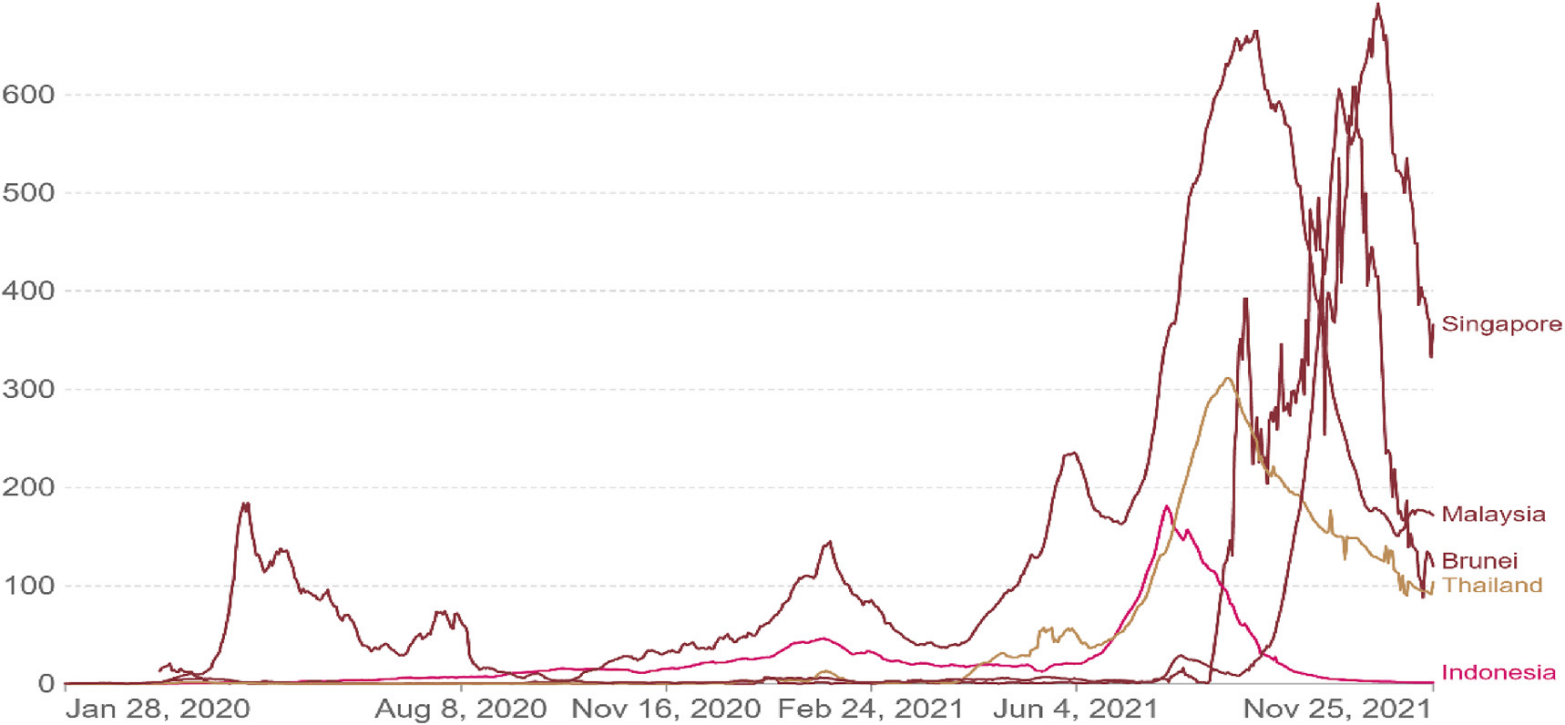
Daily new confirmed COVID-19 cases per million people in south east countries. Source (John Hopkins University, 2021)

According to Figure 1 below, COVID-19 has infected almost the entire continent since the Chinese government confirmed the first cases in January. Due to persistent outbreaks in some countries despite the vaccination of about 80% of the population, the design of innovation policies that can be easily adopted by local governments is imperative to decrease the spread of the disease (Abodunrin and Oloye, 2020). Although Indonesia has been significantly affected by COVID-19 since the confirmation of the first case on March 3, 2020, there has been a steady decline in the number of daily confirmed cases, as shown in the figure below.

As the capital city with the highest cases, Jakarta plays a major role in declining the number of new cases in Indonesia. Hence, the government innovated to prevent the virus's spread by implementing a work from home policy, social distancing, and Large-Scale Social Restrictions (PSBB). The PSBB policy includes homeschooling, restrictions on public and sociocultural events or services, and limitations on transportation modes. During the COVID-19 pandemic, the Jakarta government implemented 5 stages of PSBB policies and PPKM Levels, respectively. PSBB was implemented with strict restrictions on several aspects, such as education, public and private transportation, and events, while only allowing essential aspects like health, food, and banking sectors. The differences between Stages 1 and 5 of PSBB are based on the level and focus of restriction, while the PPKM policy has a similar idea but a looser control of the movement of people. Also, PPKM requires the local government to follow several rules made by the central government, namely limiting work in offices. This was conducted by implementing a system involving 75% Work from Home (WFH) and 25% Work from the Office (WFO). Dine-in in restaurants is also limited by 25% of the capacity, while food service via delivery or take away was allowed according to the restaurant's operating hours. The adequate enforcement of these strategies would minimize the spread of COVID-19 and balance the economic sector. Meanwhile, the broad and systematic Large-Scale Social Restriction was implemented to decrease social contact (Fornaro and Wolf, 2020). The local government also adopted a massive vaccination program to prevent the spread of COVID-19, using different levels depending on the number of cases.

The Jakarta government has been a role model for its counterparts in adopting innovative policies to prevent the spread of the pandemic. According to Parthasarathy (2020a), decision-making in policy innovation encompasses five critical elements, namely relative advantage, compatibility, complexity, trialability, and observability. The government is required to implement the most suitable element in designing an appropriate strategy for handling the pandemic (Rodriguez-Manzano et al., 2020). Since consideration of the dominant elements in innovative policymaking presents a valuable context to examine, this study aimed to evaluate the relative advantage, compatibility, complexity, trialability, and observability as the essential elements of the Jakarta government's strategy in dealing with the COVID-19 pandemic. This study is also a novel investigation because it uses the new qualitative approach to explain the more dominant elements in the innovation policies of the Jakarta government.

## 2 Literature review

### 2.1 Policy innovation theory

Conceptual innovation is an early air-hook of new ideas and a reconstruction of the applied items (Parthasarathy, 2020b). According to Edler and Fagerberg (2017), innovation must be a novelty, with newness and nature among its basic characteristics (Diercks et al., 2019). Policy innovation replaces ineffective processes that are unnecessary in tackling certain problems (Boon and Edler, 2018). Hence, innovation is used to address a particular need in some circumstances (Bayram et al., 2020) and is naturally defined as an applicably new procedure (Abi Younes et al., 2020) that is closely linked to innovative public services (Sorenson, 2018).

Political leadership, competition, and collaboration are three essential aspects that boost the implementation of public policy innovation (Torfing and Ansell, 2017). Therefore, certain actors and institutional structures support and enhance its processes (Jugend et al., 2020). Emerging innovations affect internal and external factors (Mc Fadden and Gorman, 2016), of which the latter comprises actors involved in implementing policies and generating various models (Jenkins et al., 2020). This results in a commercial or non-commercial product, service, process, or new business model (Edler et al., 2016).

Policy innovation addresses certain problems and challenges (Rodriguez-Manzano et al., 2020) because it reflects a response that emerges during implementation (Matei and Bujac, 2016; Uyarra et al., 2019). Moreover, it underpins several innovation goals that boost the country's growth, local income, health, and environment (Hall and Löfgren, 2017, Weible et al., 2020). Policy innovation follows the dynamism of challenges experienced (Coad et al., 2020) and transforms continually (Hekkert et al., 2020). It is generally implemented differently (Wojnicka-Sycz and Sycz, 2016) because policymakers need to ensure public awareness (Zhu, 2017). Furthermore, it is broad and dynamic in response to existing challenges (Bogers et al., 2018), as policies are considered to grow continuously (La et al., 2020). Transformative innovation policies are also frequently associated with enormous unpredictability (Edler and Fagerberg, 2017).

### 2.2 Implementing innovation policy during a pandemic situation

Policy innovation should be implemented adequately during a pandemic because of economic growth, clarity of the objectives to be integrated, and the minimization of the effects on the public (Rodriguez-Manzano et al., 2020). Therefore, implementing policy innovation enhances economic growth in the social, health, and finance sectors (Keane and Neal, 2020). This corresponds to the study by Nicholls (2020), which stated that policy innovation is a solution to economic sustainability. During the early period of the pandemic outbreak, approximately one-third of the countries worldwide implemented the lockdown policy to prevent the spread of COVID-19 (Rodriguez-Manzano et al., 2020). This step is comparable to the Chinese government's efforts to contain the spread of the virus. According to Dai et al. (2020), the State of China executed a rigorous approach through a lockdown policy that successfully contained the disease. The country's inventive skills were more critical in reducing the COVID-19's high-risk effect (Bayram et al., 2020), revealing that other governments require innovation through policy development that is responsive to the situation (Grillitsch et al., 2019). Meanwhile, the Jakarta government classified a large-scale social restriction (PSBB) into five phases and developed a strategy to mitigate the COVID-19 spread at the PPKM level (Agni et al., 2021).

PSBB is a limitation of certain activities of residents in an area suspected of contamination or infection to prevent the possibility of spreading the disease (Hadning and Fauzi, 2022). The Jakarta government enacted five stages of PSBB and PPKM policies each (Agni et al., 2021). Basically, PSBB imposed stringent limitations on various areas, including education, public and private transportation, alongside events (Dewi et al., 2021). The distinctions between Stages 1 and 5 are in the degree of limitation and the sector restriction's emphasis (Tri Sakti, Mohamad, and Azlan, 2021). In the first stage, the government advised the suspension of activities in offices, houses of worship, public places, facilities, and transportation and instructed employees to work from home (Pardamean et al., 2021). The second stage involved a similar suspension with stricter restrictions involving travel (Trisandy et al., 2021). In the third stage, the restrictions were loose, especially on offices and transportation. Work activities were allowed to continue with a 25% limit on the number of people in an office building at any given time, while public transport was to operate with a 50% limit on the maximum number of passengers (Hutahayan, 2021). In the fourth stage, some restrictions were eased to revive the economy, such as offices, education, and transportation aspects, by allowing the operation of an offline scheme while adhering to the protocol (Megananta and Fauzi, 2022). The fifth stage involved the imposition of more strict restrictions on office activities, entertainment, and public transportation. Office activities were conducted at home unless there was a vital interest, while places of worship, entertainment, and the tourism sector were shut down. Additionally, activities involving several people in public places were postponed, though dine-in restrictions were not imposed on food businesses, which only accepted take-away orders to limit public transportation (Afdhal et al., 2022). In the PPKM stage, the government imposed more loose restrictions, as activities were not banned but rearranged to avoid the production of new clusters and an increase in positive COVID-19 cases. Unlike PSBB, there was no distinction between the first and fifth stages of PPKM, and the level only explained the time of implementation. During the PPKM stage, the government allowed construction activities to operate at 100% with the implementation of stricter health protocols. Furthermore, a 50% capacity limit was imposed on places of worship, public transportation was allowed to operate by strictly following the protocol, while workplaces with the WFO scheme functioned at 75% (Sumadhinata et al., 2021).

**Figure 2:**
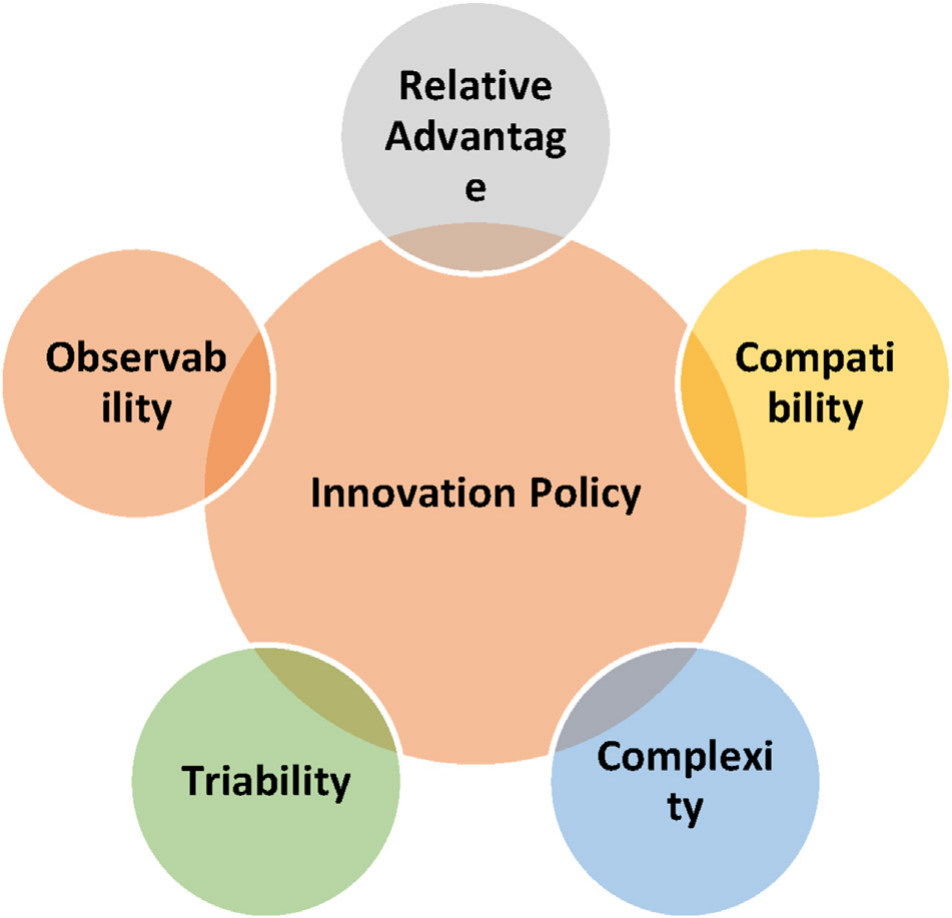
Theoretical framework. Source: Diercks et al. (2019).

COVID-19 has affected several sectors in nearly all countries (Rincón-Aznar et al., 2020), as even the China cases show that a pandemic threat places enormous pressure on all sectors (Gershon et al., 2020). During the early pandemic, almost all countries implemented policies to slow the disease transmission (Ali and Alharbi, 2020), which effectively suppressed the spread of COVID-19 but caused a global economic and social crisis (Fernandes, 2020). As shown in Figure 2, the innovation policy encompasses five important elements, including relative advantage, compatibility, complexity, trialability, and observability (Diercks et al., 2019). Therefore, the government must adopt the most suitable element in designing an innovation policy that is appropriate for the situation and can effectively address certain problems and challenges (Rodriguez-Manzano et al., 2020).

**Figure 3:**
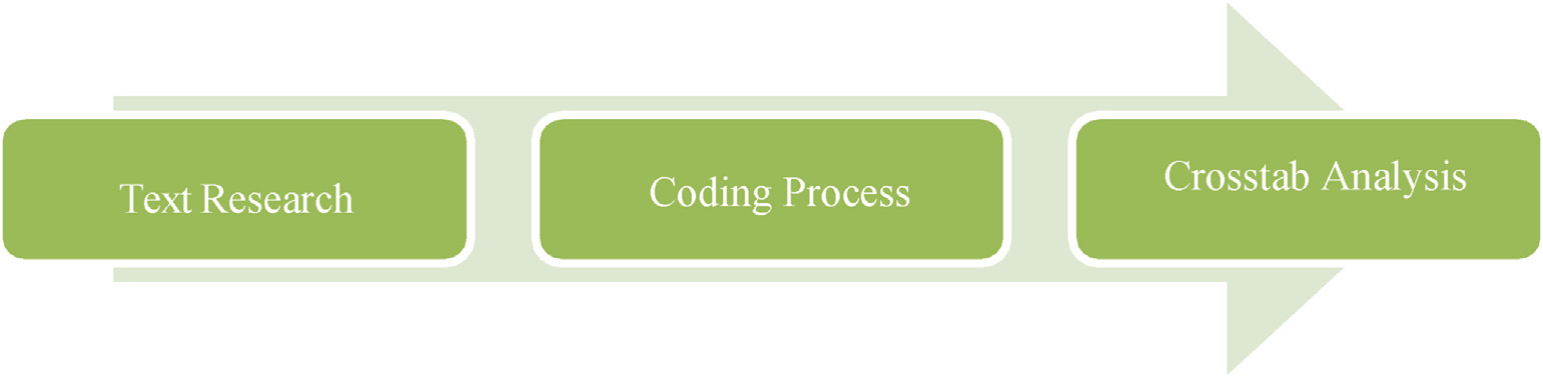
Analysis process of the research.

A relative advantage is a policy innovation with numerous benefits and greater values (Farrugia and Plutowski, 2020, Schwanen, 2018). Meanwhile, compatibility or suitability involves the ability of two or more properties to co-exist, ensuring that the former creations are not abandoned based on huge cost factors, thereby highlighting their role in transition processes (Grillitsch et al., 2019). Compatibility facilitates the adaptation process and ensures rapid and precise implementation of innovations (Uyarra et al., 2019). Conversely, complexity implies that a policy needs to be more complex than the previous innovation. These rules should be intensified to reduce the pandemic's impact, such as the rise in the prices of basic commodities (Abodunrin and Oloye, 2020). Another element, trialability, involves applying the implemented policy after being tested and proven to have added more value than the old strategy. This indicates an innovative product needs to pass the public test stage for its quality to be evaluated (Kuhlmann and Rip, 2018). Finally, observability implies a regulation whose function is easily observed and produces a better outcome. Policy innovation changes easily based on adaptation to these challenges (Bayram et al., 2020).

## 3 Method

This qualitative study employed the Computer-Assisted Qualitative Data Analysis Software (CAQDAS) method, named the NVivo application (Buchanan and Jones 2010; Butsaban 2020; Cole and Rioufreyt, 2020). The purposive sampling technique was used to collect data from 40 articles obtained from four online media sources that provide information about the COVID-19 social restriction policy in DKI Jakarta. These sources, namely Detik.com, Kompas.com, Liputan6.com, and Kumparan.com, were considered based on the neutrality of the information published. Subsequently, the data were analyzed using the NVivo application, while validity was confirmed via a source test (AlYahmady and Al Abri, 2013).

The Computer-Assisted Qualitative Data Analysis method was used because it generates data in the form of text and media (Woods et al., 2016). NVivo has been utilized in qualitative studies for almost 25 years to assist with unstructured social media data and online news (Paulus et al., 2017). According to Edwards-Jones (2014), NVivo helps organize, analyze, and discover insights associated with unstructured or qualitative data, including interviews, open survey responses, journals, articles, social media, and web content (Edwards-Jones, 2014). Also, Woods et al. (2016) stated that results are obtained by coding several innovative indicators, followed by displaying a tabulated Crosstab and Cluster Analysis that shows the number of gratuities.

Meanwhile, the first step in data collection involved searching for online articles and data from news publications about PSBB policies, while the second step determined a scheme code based on the theory used. The content of news articles on a scheme code was analyzed using a text-mining tool or code, while a crosstab analysis was used to compare the elements of the innovation policy and visualize the percentage comparison. The X-axis was classified into four nodes based on the theory of innovation policy. The X-Axis scheme nodes were classified into a relative advantage, compatibility. complexity, trialability, and observability. Further, the y-axis was classified into stages of PSBB policies and PPKM policies. Cluster analysis has aim to analyze the correlation between nodes and other nodes (Woods et al., 2016). Crosstab analysis helps the researcher to find the percentage through a coding scheme based on the theory used. Furthermore, Cross-tabulation analysis, also known as contingency table analysis, is most often used to analyze categorical (nominal measurement scale) data (Sotiriadou et al., 2014). As a statistical analysis method that allows categorical evaluation across a data set, cross-tabulation can help to uncover variables or multiple variables that are based on scheme codes (Dollah et al., 2017). see (Figure 3).

## 4 Result

**Figure 4:**
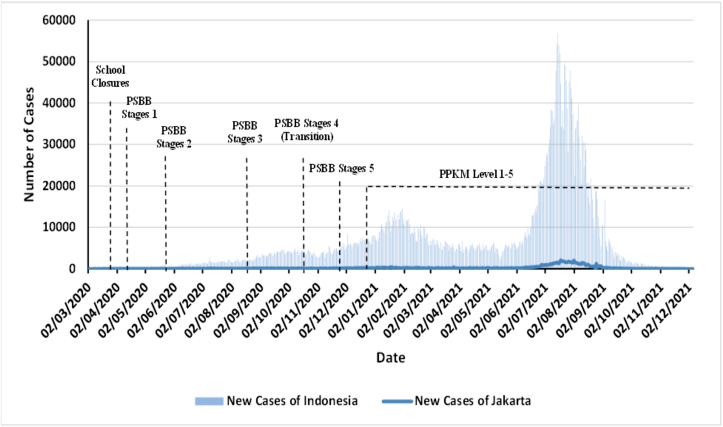
The timeline of the social scale restriction (PSBB) policy of DKI Jakarta.

The Ministry of Health permitted the first PSBB policy from April 10 to 24, 2020, in Jakarta, the province with the highest COVID-19 infection in Indonesia. However, the number of active cases increased at the end of the first PSBB, as shown in Figure 4. This caused the Governor of Jakarta to formulate the second and third stages, involving the TNI Polri for surveillance and imposing sanctions on the law violators. The second PSBB implementation was extended to determine DKI Jakarta's functionality during the New Normal and was re-implemented in September 2020 due to the sudden surge in positive COVID-19 cases. This policy is currently referred to as the Strict PSBB.

**Figure 5:**
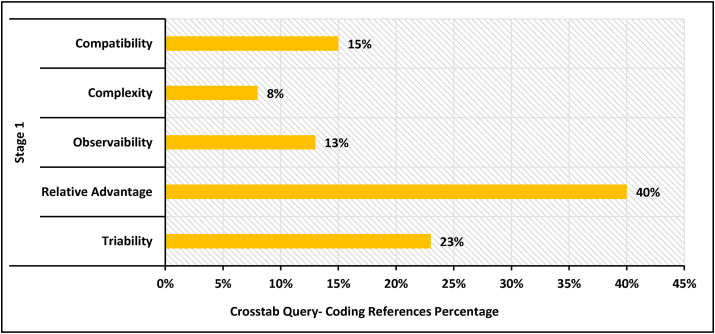
Crosstab analysis of the policy innovation element in stage 1.

Figure 5 shows that the first PSBB stage in Jakarta indicated 23% for trialability, 40% for relative advantage, 15% for observability, 8% for complexity, and 15% for compatibility. Trialability signifies that the innovation was tested and proved profitable, while relative advantage denotes its economic and social benefits. According to Anies Baswedan, DKI Jakarta Province was the first city in Indonesia to enforce the PSBB regulation for handling COVID-19. Subsequently, the PSBB innovations were more advantageous and added greater value than the previous policies. Governor Anies stated that the first stage of the PSBB implementation had effectively curbed the pandemic spread in DKI Jakarta (Mahendra, 2020). The policy involved a dramatic decrease in passengers on public transport, including Trans Jakarta and MRT, which respectively conveyed 950,000 to 1 million persons and 80,000 to 90,000 persons daily. These figures were reduced to 91,000, approximately 9% of the previous value, and 5,000 or 5%, respectively. Similarly, the LRT carries approximately 200 people per day (Syafri et al., 2020).

Anies Baswedan stated that the government would force citizens and businesses to comply with the PSBB provisions outlined in the Jakarta Governor Regulation No. 33 of 2020. The Governor imposed a large-scale social scale restriction policy in the stage 1 scheme to ensure the economic sectors functioned effectively and contained the COVID-19 cases to relative advantage. According to this regulation, all office activities were to be discontinued or conducted at home.

As most people did not understand the health implications of COVID-19 during the first PSBB policy stage, the governor instructed his ranks, including RT and RW, to massively disseminate the guidelines to the villagers constituting the city administration. This was performed to ensure residents remained at home, washed their hands frequently, kept a safe distance, and wore nose masks when leaving their houses. Furthermore, the governor's party, the police, and TNI conducted education programs for the residents and companies during the regulation's enforcement. Although Anies believed that the PSBB helped curb the spread of COVID-19, 14 days of the policy implementation were insufficient to control the spread of the coronavirus in the city, forcing an extension of the regulation. The residents were expected to comply with the PSBB rules as they were educated by the Jakarta government through massive socialization and law enforcement officials, alongside the DKI Province implemented strict sanctions on violators.

**Figure 6:**
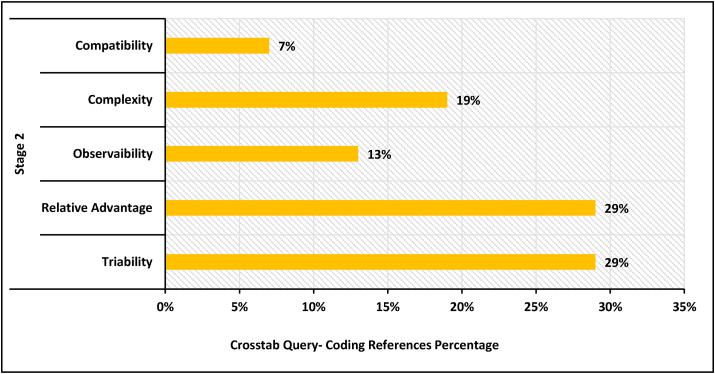
Crosstab analysis of the policy innovation element in stage 2.

Figure 6 shows the results of the PSBB second stage, indicating 37% trialability, 25% relative advantage, 7% observability, 25% complexity, and 3% compatibility. This stage was implemented from April 24 to May 22, 2020, and the results revealed that trialability is the most dominant aspect, followed by relative advantage and complexity. Stage 2 involved a higher complexity because most citizens failed to abide by the large-scale social restrictions in stage 1. Therefore, the Jakarta government designed stricter policies on the large-scale social restrictions in Stage 2 through an innovation that entailed a higher degree of complexity than the previous policies, offering an improved outcome.

An evaluation was conducted during the implementation of the first stage to suppress the viral spread. The decision to extend PSBB by 28 days, from April 24 to May 22, 2020, was based on Anies' discussion with experts in infectious diseases and the DKI Health Office. In the second stage, regulations were established to restrict entry into the city after Eid. This was in line with President Joko Widodo's appeal against traveling home, concerning which Anies stated that residents who traveled to their hometown could not return to the city anytime soon. Although the number of positive cases decreased, the strictness of the PSBB rules was assured. Hence, the recent decline in positive cases does not imply the regulation's leniency but the discipline and adherence of all parties to the regulation.

**Figure 7:**
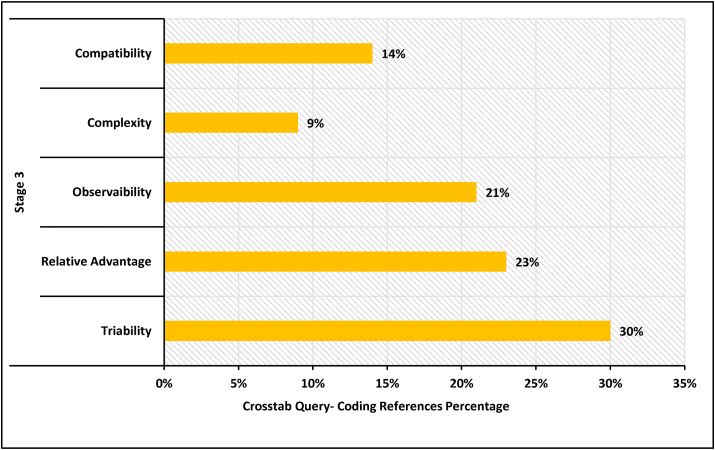
Crosstab analysis of policy innovation element on stage 3.

Stage 3 results show that policy innovation was 30% for trialability, 23% for relative advantage, 20% for observability, 9% for complexity, and 14% for compatibility. Figure 7 shows the dominance of trialability and relative advantage, though different dominant values were obtained in the third rank. In this stage, the third dominant value was observability, indicating that innovation should be observable in functionality and the generation of an effective outcome. After conducting the first and second PSBB stages, the third stage was improved, with no provisions or relaxations for activities that caused a spike in Covid-19 cases.

**Figure 8:**
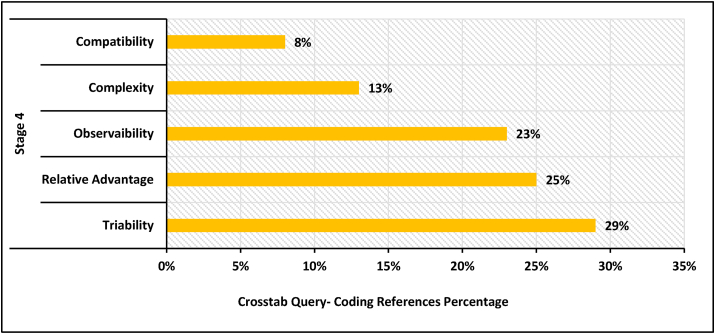
Crosstab analysis of the policy innovation element in stage 4.

Figure 8 shows that the large-scale social restrictions in stage 4, known as the transition PSBB, indicated 29% trialability, 25% relative advantage, 23% observability, 13% complexity, and 8% compatibility. The dominant aspect was similar to the third stage, differing only in the percentage. Relative advantages and trialability were the dominant elements that determined the implementation of a scheme in PSBB stage 4. The trialability aspect of the fourth stage was shown by centering the transitional PSBB on the New Normal. Stage 4 was applied to determine the progress towards the new normal, and the relative advantage aspect was reflected in the relaxation of some restrictions to revive the economy. The "New Normal" resulted from the numerous implementations of these regulations. Conversely, the observability aspect motivated the decision to ease some restrictions, permitting workplaces to operate an offline scheme while adhering to the protocol.

**Figure 9:**
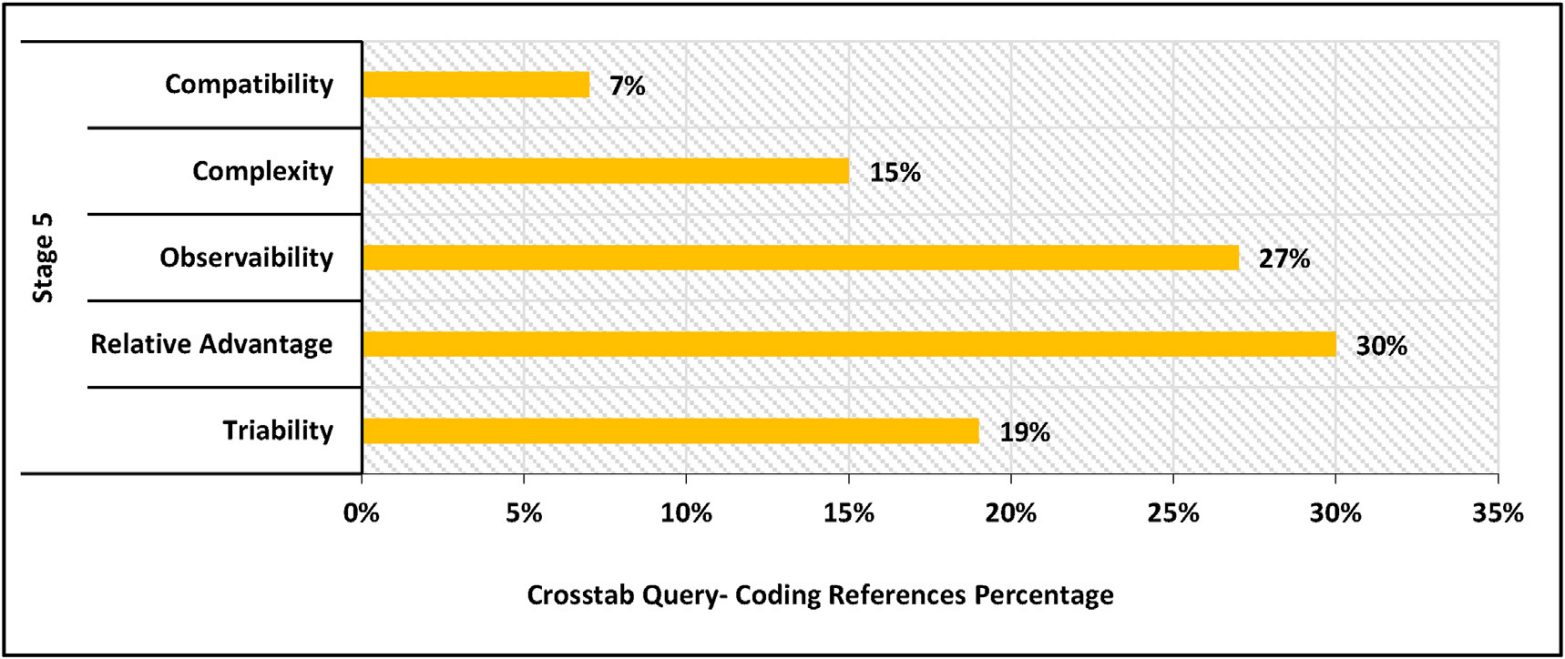
Crosstab analysis of the policy innovation element in stage 5.

Figure 9 shows the results from PSBB stage 5, indicating 19% trialability, 30% relative advantage, 27% observability, 15% complexity, and 7% compatibility. These results were different because the dominant aspects were relative advantage and observability before triability. Observability and relative advantage were depicted through the actions of Anies Baswedan by re-applying the strict PSBB, which was advantageous over the transitional regulation. This is because the strict policy promoted social restrictions, including office activities, entertainment, and public transportation. As a result, office activities were scheduled to be conducted at home unless there was a vital interest, while places of worship, entertainment, and the tourism sector were shut down. Activities involving several people in public places were also postponed, though dine-in food businesses were allowed to accept only take-away orders, thereby restricting public transportation.

**Figure 10:**
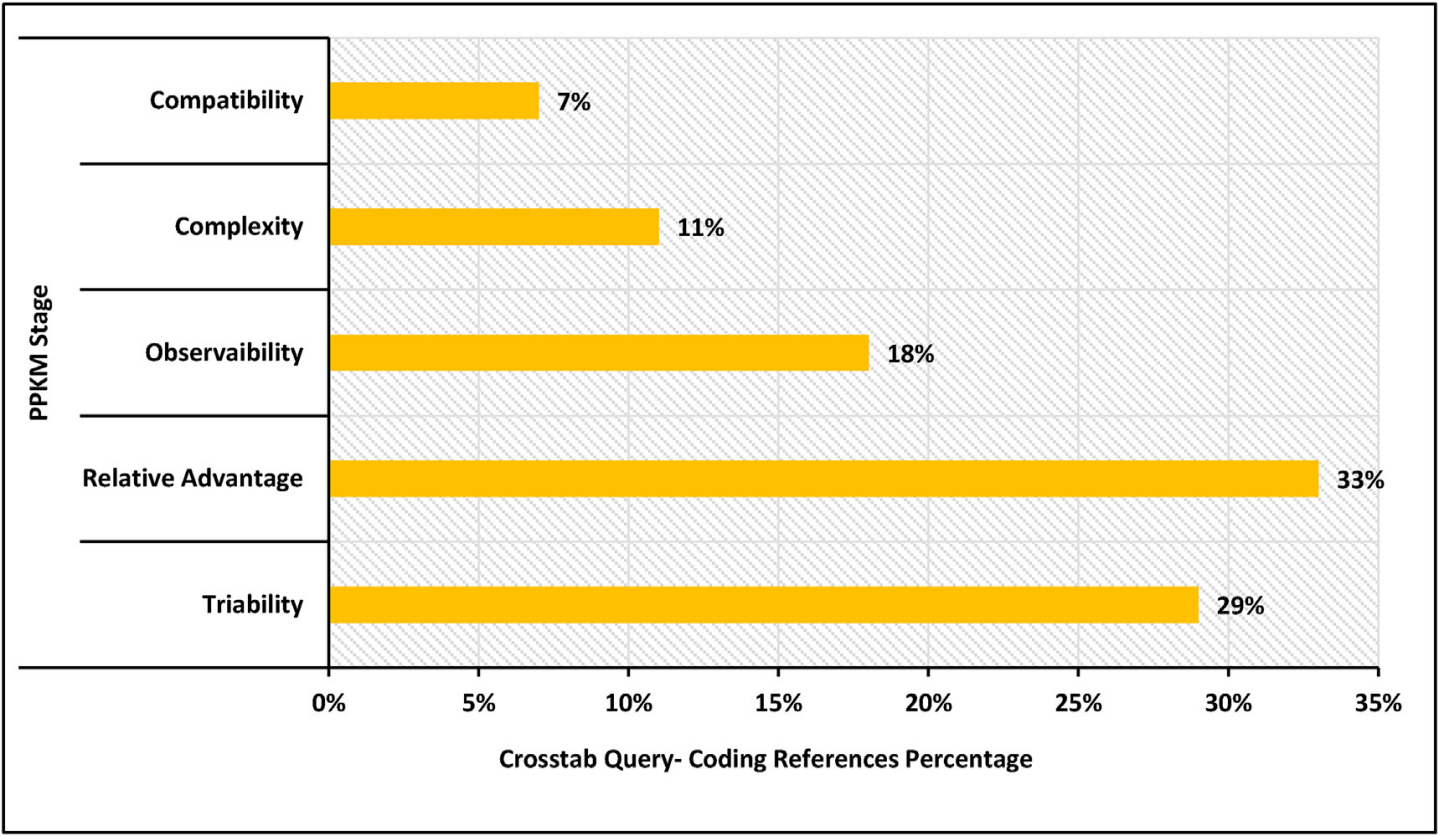
*Crosstab Analysis of the Policy Innovation Element in the PPKM Stage*.

**Figure 11:**
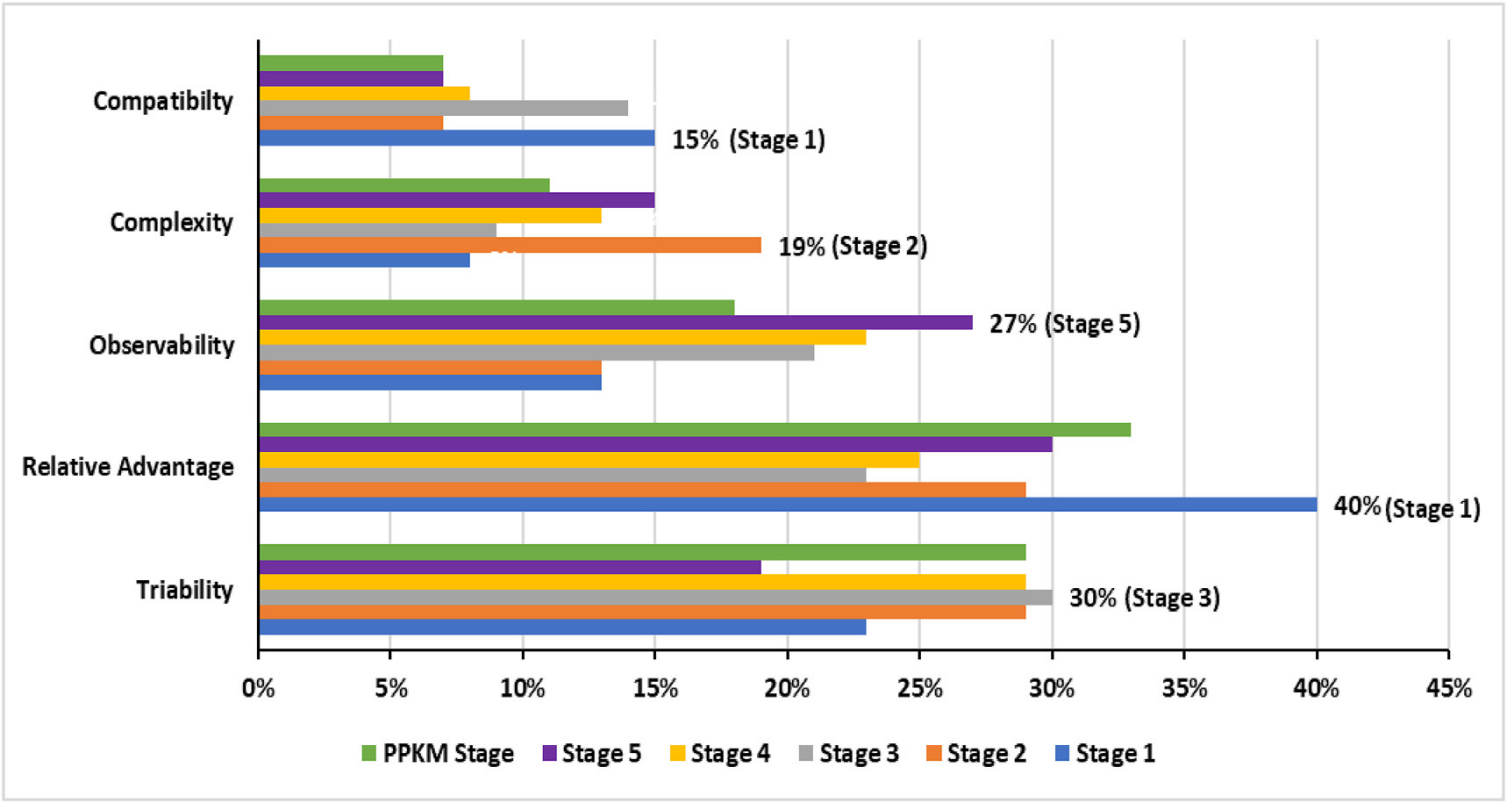
*The Domination Comparison of the Innovation Policy Elements in PSBB Stage 1-5 and PPKM stage*.

Figure 10 shows the results of the PPKM stage, indicating the dominance of relative advantage at 33% and trialability at 29%. The Jakarta government's desire to curb the number of COVID-19 cases decreased, prompting the imposition of this policy because of the ASEAN culture of celebrating the New Year. Therefore, the Jakarta government aimed to prevent an increase in cases and abide by the President's instruction to tighten the restrictions. The PPKM policy required offices in non-essential sectors to implement the Work from Home (WFH) scheme at a 100% rate, while essential sectors conducted 50% of their businesses from an office. Employees in critical sectors, such as energy, health, security, logistics, and transportation, as well as food and beverage, were allowed to WFO by implementing strict health protocols. Other essential sectors included supporting industries, petrochemicals, cement, vital national objects, disaster management, national strategic projects, basic utility construction, and industries fulfilling basic community needs. Consequently, this stage impacted the economy because shopping centers, malls, and trade centers were temporarily closed, as the government tightened the restrictions from December to January (see Figure 11).

The line diagram above shows the comparison between the implementations of PSBB 1, 2, 3, 4 (Transition), and 5 (Tight). According to the coding in Figure 10, trialability was highest at stage 3 of Transitional PSBB, while the least was recorded at stage 5. The relative advantage was highest at the first and PPKM stages, complexity was highest and lowest at PSBB 2 and 1, while observability was highest and lowest at stages 5 and 1, respectively. Conversely, the highest compatibility scores were observed at stage 1. The PSBB Stage 1–4 policies and PKKM policies become unique policies as a new approach to adapt to unpredictable situations like the COVID-19 situation. The relationship between PSBB Stage 1–4 policies and PPKM policies shows that to prevent pandemic situations the government should consider the several impacts that appear due to the pandemic situation. The emphasis of the policy scheme should not be carried out equally. The government should be considering the conditions in each place. A comparison of PSBB and PPKM policies by the Jakarta government shows that to prevent COVID-19 in developing countries should implement a new approach considering the informal sector becomes an important sector to move the wheels of the economy. The implemented PSBB and PPKM policies as a new solution to prevent the spread of COVID-19 compared to implementing lockdown policies to prevent COVID-19 has a positive impact to maintain the negative effect on social and economic aspects.

## 5 Discussion

Following approval by the minister of health, the Jakarta government created a policy to reduce the COVID-19 transmission. This was expected to reduce and control the spread of the pandemic, which has promoted innovation in its handling (Rodriguez-Manzano et al., 2020). The government designed a scheme to reduce the risk of impact and erected barriers to prevent the viral spread (Bayram et al., 2020), denoting that innovation was needed to formulate a policy based on the emerging situation (Nicholls, 2020). According to Edler and Fagerberg (2017), innovation must be a novelty and the newest development regarding the conditions (Edler and Fagerberg, 2017). Implementing a policy innovation would replace the processes that are ineffective in tackling certain problems (Grillitsch et al., 2019) and resolve a particular need during unpredictable situations (Kuhlmann and Rip, 2018). This would be an important government action because of the rapidly changing situation (Bogers et al., 2018), considering the quick-spread nature of the COVID-19 virus. Therefore, innovative capabilities must be maintained in order to design a dynamic strategy according to existing challenges (Gunner et al., 2020).

During this extremely precarious situation, the Jakarta government implemented a large-scale social restriction policy divided into five stages to prevent a surge in COVID-19 cases (Azoulay and Jones, 2020). This included Governor Regulation Number 47 of 2020 concerning restrictions on travel activities outside the DKI Jakarta Province to limit the virus's spread. Consequently, Jakarta's entire population was prohibited from traveling outside the province to minimize the risk of increased contamination, using an innovative strategy that effectively maintained economic and social impact.

The pandemic has caused new problems in various life aspects, particularly the economy (Gershon et al., 2020). During the early outbreak, around a third of the countries implemented the lockdown policy to prevent the spread of COVID-19 (Rodriguez-Manzano et al., 2020). This action is similar to the Chinese government's measures to handle the disease, as according to Dai et al. (2020), the state implemented a strict strategy through a lockdown scheme that effectively controlled the viral spread. However, the lockdown scheme negatively affected the economy, especially the industrial sector, because the facilities were closed for a long time in many countries (Heinonen and Strandvik, 2020, Keane and Neal, 2020). This significantly affected developing countries (Zhu, 2017), whose economic capability was insufficient in curbing the pandemic (Abbas et al., 2020).

Currently, many countries are innovating their policies to curb the spread of COVID-19 (Ali and Alharbi, 2020). The study by La et al. (2020) mentioned four stages employed by Vietnam for the prevention of COVID-19. First, the policy focused on assessment, while the second stage was centered on minimizing the spread by taking precautionary measures, travel restrictions, and market control. The third stage aimed to impose stricter measures, and the fourth stage also entailed several strict rules, such as the temporary suspension of issuing visas to foreigners. In South Korea, the strategy was based on relevant, innovative developments, consisting of quarantine, contact tracing, rapid testing, and the transparent disclosure of all data, including drives and walk-throughs (Lee and Choi, 2020). Regarding these policies, a few countries are focusing on designing a scheme that other governments can easily adopt (Kimura et al., 2020). In line with this, the Jakarta government created a policy that the local governments could easily adopt. This is a positive development, as decreasing the COVID-19 spread requires collective action (Torfing and Ansell, 2017), which can be achieved through collaboration between local and central governments (Boon and Edler, 2018).

## 6 Conclusion

The government's innovative capabilities are important in mitigating the high-risk impact of COVID-19 and this requires the formulation of a policy based on the emerging situation. Consequently, the Jakarta government divided a large-scale social restriction (PSBB) into five stages and designed a policy to decrease the COVID-19 transmission via the PPKM stage. This study revealed that trialability was highest at stage 3 or the Transitional PSBB and lowest at stage 5, while relative advantage was highest and lowest at the first and PPKM stages, respectively. Conversely, complexity was highest and lowest at PSBB 2 and 1, observability was highest and lowest at stages 5 and 1, respectively, while the highest compatibility scores were at stages 1. The relative advantage aspect was dominant in the designed COVID-19 scheme, though the Jakarta government also implemented trialability. Hence, the two aspects of the policy are being used to simplify the adoption of innovations for local governments to slow the spread of COVID-19.

The pandemic also promoted innovation in handling the viral spread through the design of a scheme to face unpredictable situations, reduce the impact of the disease, and erect barriers to prevent further contamination. Jakarta's innovation strategy effectively maintains the economic and social impact for developing countries, whose capabilities are ordinarily insufficient in curbing the pandemic**.** Therefore, some governments are focusing on innovating a policy design scheme that would be easier to adopt. A collaboration between the local and central governments is also critical in slowing the COVID-19 spread, as it requires collective action.

### 6.1 Limitations and implications

There were limitations and obstacles during the content analysis due to the management of unstructured data, and caution was needed while collecting data to obtain online news articles relevant to the study question. Furthermore, this study covered only Jakarta as one of the capital cities in the South East that successfully dealt with COVID- 19. The findings also contrasted with previous investigations which stated a lockdown as one of the best ways to handle the pandemic because this scheme would be difficult for developing countries to implement. COVID-19 has exerted pressure on countries and a lockdown scheme would imply the need to boost the economic sector. Practically, developing countries could innovate their policies based on the field situation, and relative advantage, as well as trialability, can assist in controlling the number and economic impact of COVID-19 cases.

## Declarations

### Author contribution statement

Retnowati WD Tuti: Conceived and designed the experiments; Performed the experiments; Analyzed and interpreted the data; Wrote the paper.

Achmad Nurmandi: Conceived and designed the experiments; Analyzed and interpreted the data; Contributed reagents, materials, analysis tools or data; Wrote the paper.

Abitassha Az Zahra: Performed the experiments; Analyzed and interpreted the data; Contributed reagents, materials, analysis tools or data.

### Funding statement

This research did not receive any specific grant from funding agencies in the public, commercial, or not-for-profit sectors.

### Data availability statement

Data included in article/supplementary material/referenced in article.

### Declaration of interests statement

The authors declare no conflict of interest.

### Additional information

No additional information is available for this paper.
